# Supplementary material for: Mutations in PCYT2 disrupt etherlipid biosynthesis and cause a complex hereditary spastic paraplegia
Source: Brain. 2019 Oct 22;142(11):3382–97. doi: 10.1093/brain/awz291 (PMC6821184; doi:10.1093/brain/awz291)
Supplement: awz291_Supplementary_Data [file awz291_supplementary_data.zip › awz291-Suppl_data/Supplementary_Data4.zip!/Supplementary information 4 - plasma lipidomics.zip!/Plasma lipidomics/Control vs Patient/Statistical plots and data/SPH species/Random forest.pdf]

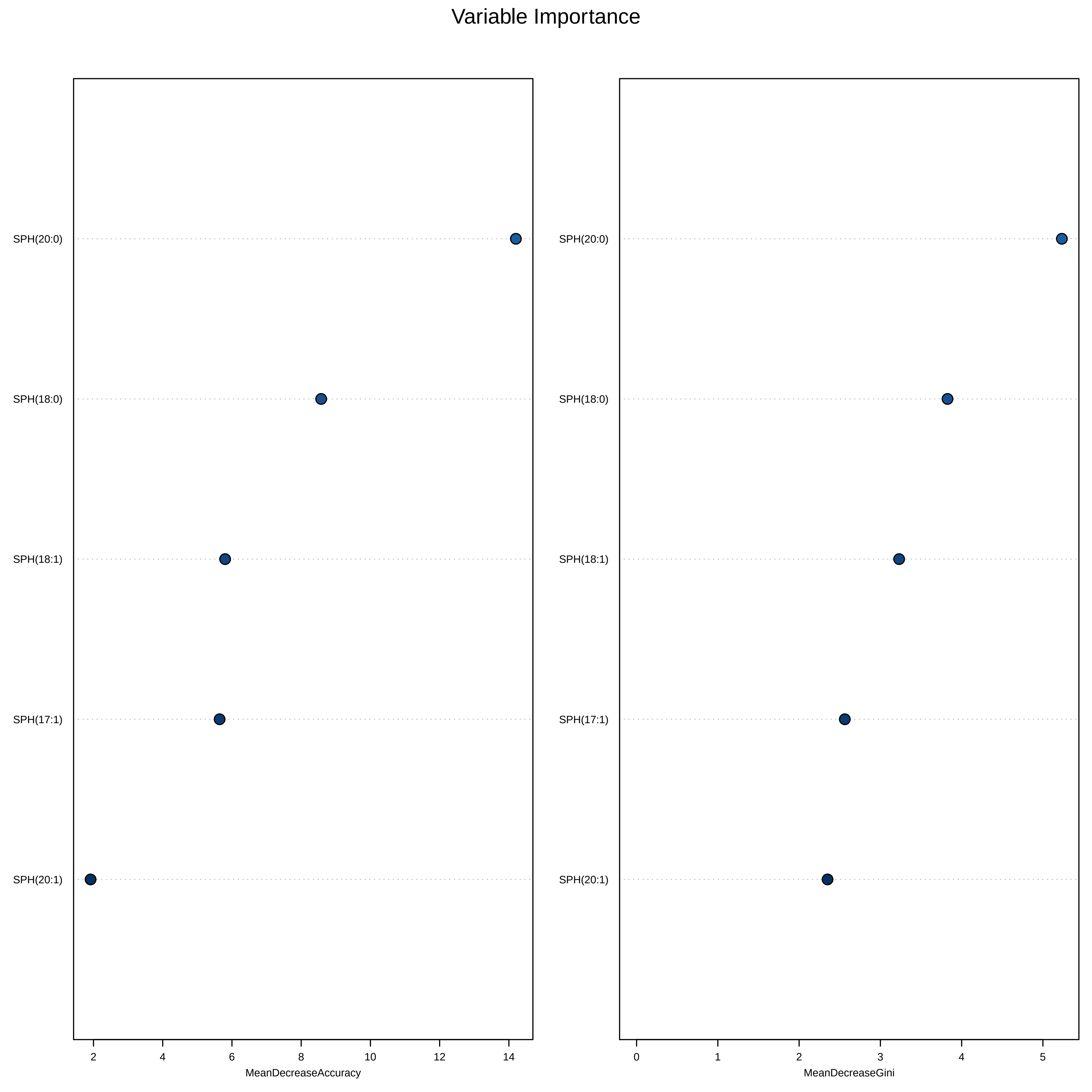
Variable Importance
SPH(20:0)
SPH(18:0)
SPH(18:1)
SPH(17:1)
SPH(20:1)
2
4
6
8
10
12
14
MeanDecreaseAccuracy
0
1
2
3
4
5
MeanDecreaseGini
